# Succinate Overproduction: A Case Study of Computational Strain Design Using a Comprehensive *Escherichia coli* Kinetic Model

**DOI:** 10.3389/fbioe.2014.00076

**Published:** 2015-01-05

**Authors:** Ali Khodayari, Anupam Chowdhury, Costas D. Maranas

**Affiliations:** ^1^Department of Chemical Engineering, The Pennsylvania State University, University Park, PA, USA

**Keywords:** computational strain design, kinetic model, bilevel optimization, succinate overproduction, model parameterization

## Abstract

Computational strain-design prediction accuracy has been the focus for many recent efforts through the selective integration of kinetic information into metabolic models. In general, kinetic model prediction quality is determined by the range and scope of genetic and/or environmental perturbations used during parameterization. In this effort, we apply the k-OptForce procedure on a kinetic model of *E. coli* core metabolism constructed using the Ensemble Modeling (EM) method and parameterized using multiple mutant strains data under aerobic respiration with glucose as the carbon source. Minimal interventions are identified that improve succinate yield under both aerobic and anaerobic conditions to test the fidelity of model predictions under both genetic and environmental perturbations. Under aerobic condition, k-OptForce identifies interventions that match existing experimental strategies while pointing at a number of unexplored flux re-directions such as routing glyoxylate flux through the glycerate metabolism to improve succinate yield. Many of the identified interventions rely on the kinetic descriptions that would not be discoverable by a purely stoichiometric description. In contrast, under fermentative (anaerobic) condition, k-OptForce fails to identify key interventions including up-regulation of anaplerotic reactions and elimination of competitive fermentative products. This is due to the fact that the pathways activated under anaerobic condition were not properly parameterized as only aerobic flux data were used in the model construction. This study shed light on the importance of condition-specific model parameterization and provides insight on how to augment kinetic models so as to correctly respond to multiple environmental perturbations.

## Introduction

Engineered microorganisms are increasingly being used as cellular factories for the bio-production of chemicals of interest (Curran and Alper, 2012; Hong and Nielsen, 2012; Lee et al., 2012). Keeping pace with genome editing techniques for strain design, several computational tools have been developed to identify system-wide genetic modification strategies that improve the yield of targeted biochemicals (Pharkya et al., 2004; Kim et al., 2011; Xu et al., 2011; Maia et al., 2012; Cotten and Reed, 2013a). In general, these tools rely on a stoichiometric representation of a metabolic network and solve bilevel optimization problems to suggest prioritized intervention strategies that divert metabolic flux towards the chemical of interest (Segre et al., 2002; Burgard et al., 2003; Kim and Reed, 2010; Rocha et al., 2010; Tepper and Shlomi, 2010). The methodology and comparative benefits of each procedure is discussed in detail elsewhere (Zomorrodi et al., 2012). However, key methodological impediments of these approaches are the stoichiometry-only representation of metabolism and the on–off representation of regulation. This may lead to a metabolite concentration, enzymatic activity, and metabolic regulation-agnostic intervention strategies. Therefore, identified flux re-direction predictions (especially up/down flux modulation) are sometimes difficult to translate into actionable genetic interventions. For example, it is unclear if a desired metabolic flux up-regulation is achievable or even consistent with enzyme kinetics or physiological metabolite concentrations.

Some of the shortcomings of genome-scale stoichiometric models in quantifying the effect of concentration and enzyme levels on reaction throughput and regulation can be addressed by kinetic models of metabolism (Mahadevan et al., 2002; Fleming et al., 2010; Jamshidi and Palsson, 2010; Smallbone et al., 2010; Feng et al., 2012). Kinetic models yield a system of ordinary differential equations (ODEs) that describe the time evolution of metabolite concentrations, enzyme activities, and reaction fluxes. Several efforts have been made in recent years for improving the accuracy of stoichiometry-based tools by partially integrating kinetic information (Nikolaev, 2010; Song and Ramkrishna, 2012; Angermayr and Hellingwerf, 2013; Almquist et al., 2014). However, most of these procedures are aimed towards improved metabolic phenotype prediction through *ad hoc* constraints (Cotten and Reed, 2013b) rather than strain design. The k-OptForce procedure (Chowdhury et al., 2014) extends the previously developed strain-design OptForce algorithm (Ranganathan et al., 2010) by integrating all available mechanistic details afforded by kinetic models within a constraint-based optimization framework tractable even for genome-scale models. Reactions with available kinetic descriptions yield (generally unique) steady-state flux values while the remaining reactions are only constrained by stoichiometric relations. Genetic intervention strategies consistent with restrictions imposed by maximum enzyme activity, bounds on metabolite concentrations and kinetic expressions are identified using a bilevel Mixed Integer Nonlinear Program (MINLP) optimization framework (Chowdhury et al., 2014). Examples addressed in Chowdhury et al. (2014), however, accounted for only a handful of reactions with kinetic expressions.

In this paper, we apply k-OptForce procedure for the recently published large-scale kinetic model of *E. coli* core metabolism (Khodayari et al., 2014). The kinetic model includes 138 reactions, 93 metabolites, and 60 substrate-level regulatory interactions and accounts for glycolysis/gluconeogenesis, pentose phosphate (PP) pathway, TCA cycle, major pyruvate metabolism, anaplerotic reactions, glyoxylate shunt, Entner–Doudoroff (ED) pathway, and a number of reactions in other parts of the metabolism. The model was parameterized using the ensemble modeling (EM) formalism (Tran et al., 2008) by simultaneously satisfying normalized flux data per 100 mmol of glucose uptake (for approximately 25 reactions per mutant) for the wild-type and seven single gene deletion mutants, under aerobic condition (Ishii et al., 2007). The EM approach decomposes all reactions into elementary steps bypassing the need of detail kinetic expressions. First, an ensemble of kinetic models is generated by uniformly sampling reaction reversibilities and enzyme fractions following different time trajectories but all reaching the same steady-state flux values (Tan and Liao, 2012). Next, a Genetic Algorithm (GA) implementation is used to “swap” kinetic parameterizations between models in the ensemble so as to minimize the deviations from all set of mutant network fluxes. Models constructed using flux data for a single strain do not always perform well in predicting deletion strain metabolic phenotypes (Jouhten, 2012; Villaverde et al., 2014). Unlike stoichiometric models that could reveal physiologically relevant flux re-directions in response to perturbations by re-optimizing biomass yield, kinetic models must be endowed beforehand with all known substrate-level regulatory interactions to capture metabolic responses to genetic/environmental perturbations (Jouhten, 2012; Heijnen and Verheijen, 2013; Villaverde et al., 2014). Note that while the EM based elementary mode analysis was used for strain design in an earlier effort (Flowers et al., 2013), the limited scope of the model may fail to capture genome-scale flux re-directions.

The k-OptForce procedure (Chowdhury et al., 2014) was used to identify the minimal interventions that maximize the yield of succinate production using a hybrid kinetic (Khodayari et al., 2014) and stoichiometric *i*AF1260 (Feist et al., 2007) description of *E. coli* metabolism. Succinate was chosen as the target bioproduct as there exists numerous experimental strain-engineering studies to compare the suggestions of k-OptForce procedure (Lee et al., 2005; Cao et al., 2011; Tan et al., 2011). This study was carried out under both aerobic and anaerobic conditions to assess the fidelity of the kinetic model when used to make predictions for a different environmental condition (i.e., anaerobic) than the one parameterized for (i.e., aerobic). The goal was to quantify the reduction in prediction quality moving from aerobic to anaerobic under glucose minimal condition and suggest model modifications that remedy these shortcomings. k-OptForce recapitulated existing strategies while also pointing at promising but currently unexplored interventions. In addition, results under anaerobic condition indicate that the kinetic model needs to be re-parameterized with mutant flux information involving a reversed TCA cycle routing flux towards succinate. A number of regulatory modifications of the kinetic model are also found to be necessary to better reflect metabolic fluxes associated with anaerobic succinate production. These include activation of fermentation pathways and pyruvate formate lyase (PFL) by key regulatory proteins FNR (fumarate and nitrate reductase regulation) and ArcA (aerobic respiratory control).

## Materials and Methods

**Figure 1:**
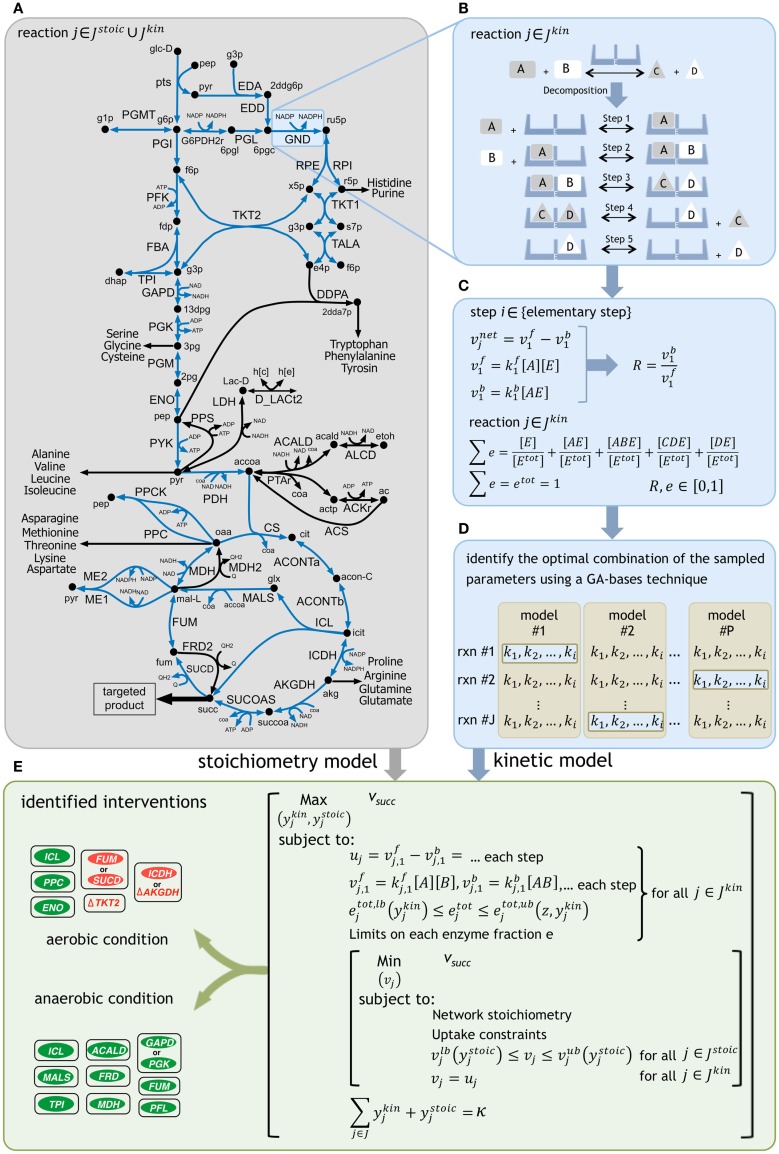
**A schematic representation of the framework**. **(A)** The reactions with kinetic descriptions are shown in blue. **(B)** The reactions are first decomposed into their elementary steps. **(C)** Elementary kinetic parameters are expressed as a function of reaction reversibilities and enzyme fractions. Reaction reversibilities and enzyme fractions are sampled to construct an ensemble of models, for any given reaction. **(D)** A genetic algorithm (GA) implementation identifies the optimal combination of the sampled parameters by minimizing the deviation from experimentally measured flux data for multiple mutant strains [see Methods of Khodayari et al. (2014)]. **(E)** The k-OptForce procedure identifies a minimal set of interventions that maximizes the yield of targeted product [see Methods of Chowdhury et al. (2014)].

Using k-OptForce, the genome-scale stoichiometry matrix is divided into two parts: reactions with stoichiometric information only (***J***^stoic^), and those having additional kinetic information (***J***^kin^). A schematic representation of the framework is depicted in Figure 1. The kinetic information was extracted from the kinetic model of *E. coli* central metabolism developed in Khodayari et al. (2014). The number of reactions in the kinetic representation is a compromise between reduction of solution space using kinetic data and run time for solving the non-linear expressions of mass conservations. Upon exclusion of the exchange/transport reactions and elimination of reactions not involved in succinate synthesis (such as glycogen pathway), a subset of the kinetic model was selected containing 36 reactions and 31 metabolites. The resulting model includes reactions from glycolysis/gluconeogenesis, PP pathway, TCA cycle, anaplerotic reactions, glyoxylate shunt, and ED pathway with available experimental data during model parameterization. This model was finally supplemented with the stoichiometric *i*AF1260 model of *E. coli* (Feist et al., 2007).

Glucose minimal condition were simulated by restricting glucose uptake flux (which serves as a basis for the fluxes in the metabolic network) to −100 mmol gDW^−1^h^−1^. Oxygen uptake was limited to −200 mmol gDW^−1^h^−1^ for aerobic condition and set to zero for fermentative condition. Regulatory information for both aerobic and anaerobic conditions was imported from the supplementary material of *i*AF1260 model (Feist et al., 2007). The minimum production levels of succinate was set at 90% of its theoretical maximum for each condition (i.e., 135 mmol gDW^−1^h^−1^ in aerobic and 149 mmol gDW^−1^h^−1^ in anaerobic conditions) while a minimum level of biomass production equal to 10% of its theoretical maximum was simultaneously imposed (i.e., 0.965 h^−1^ in aerobic and 0.303 h^−1^ in anaerobic conditions). The k-OptForce algorithm was implemented in the same stepwise procedure as described previously [see Methods in Chowdhury et al. (2014) for details]. At first, we identify all reactions that must depart (hence called MUST sets) from the reference phenotype to realize the targeted levels of overproduction of the desired chemicals under stoichiometric and kinetic constraints. Subsequently, we solve a bilevel optimization formulation (see Figure 1E) where we maximize the target flux by gradually increasing the total number (κ) of enzymatic interventions (for reactions in ***J***^kin^) and/or flux manipulations (for reactions in ***J***^stoic^) from the MUST sets. Starting from a single intervention, we stop this procedure when the target flux does not improve appreciably with additional interventions. The optimization formulations for the characterization of the overproducing network and identification of the FORCE sets were altered from the original procedure to incorporate the kinetic information of each reaction in ***J***^kin^ as a function of the decomposed expressions of its elementary steps (see Figure 1) instead of directly manipulating the reaction enzyme activities (*v*^max^). Additional constraints were imposed to express the flux of each reaction in ***J***^kin^ as the difference of the forward and reverse reactions for each elementary step. The sum of individual enzyme fractions *e* is represented by *e*^tot^ (i.e., normalized total enzyme concentration) that is equal to one in the reference (wild-type) strain, but varies when up/down-regulated in mutant strains. Here, we allowed the *e*^tot^ of each reaction to vary between zero (i.e., removal of its activity) and a 10-fold up-regulation in its expression to account for individual enzymatic perturbations in mutant strains. Likewise, the same limits of variation were set for the individual enzyme fractions *e* for each reaction.

The metabolite concentrations were allowed to vary within fivefold from their steady-state values in the reference phenotype. The FORCE set of interventions was identified in a two-step procedure [see Methods of Chowdhury et al. (2014)]. The first step identified the reactions in ***J***^kin^ (using binary variables *y*^kin^) whose enzymatic activity (i.e., *e*^tot^) must be altered from their reference level to achieve the required flux re-distribution towards succinate. The lower and upper bounds on *e*^tot^ (i.e., *e*^tot,lb^ and *e*^tot,ub^) are functions of *y*^kin^ and the maximum fold-change *z*, as follows:

$$\begin{array}{lll} e_{j}^{\mathrm{tot},\mathrm{lb}} & =\left\{\begin{array}{cc} 1,\; & \mathrm{if}\;j\in J^{\mathrm{kin}}\setminus {\mathrm{MUST}}^{L}\\ 1-y_{j}^{\mathrm{kin}},\; & \mathrm{if}\;j\in J^{\mathrm{kin}}\cap {\mathrm{MUST}}^{L}\\ \; \end{array}\right.\; & \;\\ e_{j}^{\mathrm{tot},\mathrm{ub}} & =\left\{\begin{array}{cc} 1,\; & \mathrm{if}\;j\in J^{\mathrm{kin}}\setminus {\mathrm{MUST}}^{U}\\ \left(z-1\right)y_{j}^{\mathrm{kin}}+1,\; & \mathrm{if}\;j\in J^{\mathrm{kin}}\cap {\mathrm{MUST}}^{U}\\ \; \end{array}\right.\; & \; \end{array}$$

If selected for down-regulation (i.e., when the reaction is part of MUST*^L^*), *e*^tot^ is allowed to vary from zero (*e*^tot,lb^ = 0 for *y*^kin^ = 1) to its reference expression. Otherwise, *e*^tot^ is set to one. Likewise, if selected for up-regulation (i.e., when the reaction is part of MUST*^U^*), *e*^tot^ is allowed to vary from one to a *z*-fold overexpression (*e*^tot,ub^ = *z* for *y*^kin^ = 1). The MINLP formulation for the first-step was initially solved using a local solver [DICOPT (Grossmann et al., 2002)], and the results were used as inputs to find the global optimum using the BARON solver (Sahinidis, 1996). Subsequently, by fixing the fluxes in ***J***^kin^, the second step identified additional flux manipulations in ***J***^stoic^ (using binary variables *y*^stoic^) while assuming a worst-case scenario for the inner objective function. The relation of the modified bounds $\left(v_{j}^{\mathrm{lb}},v_{j}^{\mathrm{ub}}\right)$ on the reaction fluxes in ***J***^stoic^ with *y*^stoic^ is similar to that explained for the first step of FORCE set identification for the implementation of up/down-regulations and/or reaction removals [see Methods of Chowdhury et al. (2014)].

## Results

### Examining the predictive performance of the kinetic model

The perturbed phenotype prediction accuracy of the parameterized kinetic model was first assessed for five different engineered strains under aerobic condition. The experimentally reported product yield was compared against the kinetic model and FBA predictions (see Table 1). A twofold up-regulation for small fold-change, and 10-fold up-regulation for a high fold-change are used to express enzyme up-regulation, whenever such information is not available in the relevant literature. The enzyme level manipulation in the kinetic model is achieved by changing *e*^tot^ for each particular enzyme. Gene deletions are implemented by setting the *e*^tot^ of the encoded enzyme to zero.

**Table 1:** **A comparison between model predictions and experimental yields for five different products in *E. coli* under aerobic condition**.

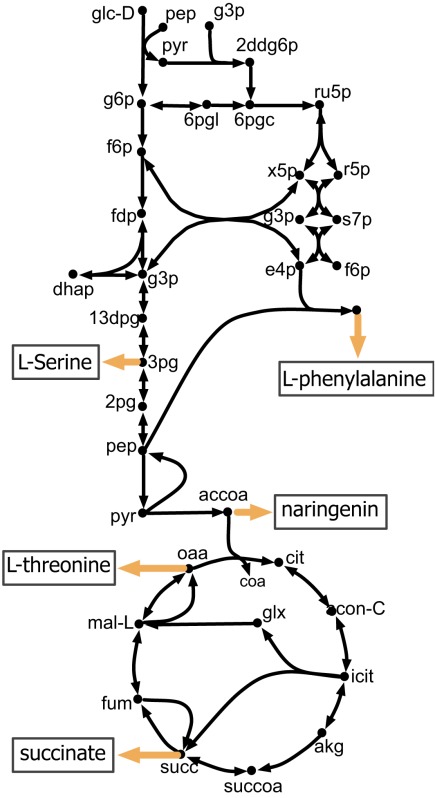	**Target product**	**Interventions with enzyme-fold-change**	**Yield (mol product/mol glucose)**		
			**FBA**	**Kinetic model**	**Experimental data**

	Succinate	ΔSUCD	0.99	0.52	0.6 (Lin et al., 2005b)
			ICL 10 ↑	
		PPC 2 ↑	
	L-serine	ΔPDH	0–0.01	0.81	0.48 (Lai et al., 2012)
		PGCD 10 ↑	
		PGK 2 ↑	
	L-threonine	PPC 2 ↑	0–0.04	0.52	0.59 (Lee et al., 2007)
		ICL 2 ↑	
	L-phenyl alanine	ΔPYK	0.44	0.11	0.36 (Baez-Viveros et al., 2007)
		DDPA 10 ↑	
		TKT1 10 ↑	
	Naringenin	ΔSUCOAS	0.43	0.07	0.11 (Xu et al., 2011)
		ΔFUM	
		ACCOAC 10 ↑	
		PDH 10 ↑	
	GAPD 10 ↑	

*The engineering strains are simulated using both the kinetic model and FBA (max biomass)*.

*SUCD, succinate dehydrogenase; ICL, isocitrate lyase; PPC, phosphoenolpyruvate carboxylase; PDH, pyruvate dehydrogenase; PGCD, phosphoglycerate dehydrogenase; PGK, phosphoglycerate kinase; PPC, phosphoenolpyruvate carboxylase; PYK, pyruvate kinase; DDPA, 3-deoxy-D-arabino-heptulosonate 7-phosphate synthetase; TKT, transketolase; SUCOAS, succinyl-CoA synthetase; FUM, fumarase; ACCOAC, acetyl-CoA carboxylase; GAPD, glyceraldehyde-3-phosphate dehydrogenase*.

**Figure 2:**
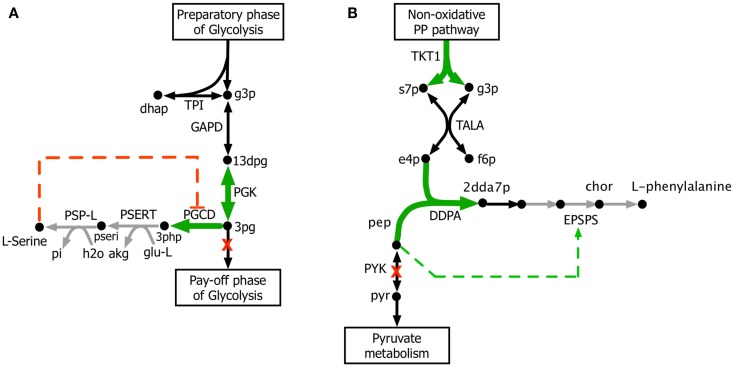
**Biosynthesis pathways for (A) L-serine and (B) L-phenylalanine**. The suggested up-regulations and knock-outs are shown with green color and red crosses, respectively. The reactions absent in the current kinetic model are shown in gray. Missing regulatory interactions (i.e., activation and inhibition) are shown with dashed lines.

The kinetic model closely matches the succinate producing strain while FBA over-estimates it because the former captures the feed-forward inhibition on glyoxylate shunt by intermediates phosphoenolpyruvate (pep) (MacKintosh and Nimmo, 1988; Ogawa et al., 2007) and isocitrate (icit) (Hoyt et al., 1988). For both L-serine and L-threonine, FBA directs all carbon flux towards biomass predicting little to no amount of product formation. The kinetic model over-estimates L-serine yield as product inhibition of the phosphoglycerate dehydrogenase (PGCD) (Grant, 2012; Li et al., 2012; Wang et al., 2014) is not captured in the kinetic model (see Figure 2A). In contrast, the kinetic model under-estimates the yield of L-phenylalanine production. A possible reason is that the feed-forward activation of pep on 5-enolpyruvylshikimate-3-phospahte synthase (EPSPS) (Gruys et al., 1992) is absent in the kinetic model (see Figure 2B). In addition, due to lack of experimental data during parameterization, the model over-estimates the inhibitory effect of phosphate on transaldolase (TALA) activity (Sprenger et al., 1995), which further restricts flux towards l-phenylalanine production. The naringenin engineered strain productivity is better reflected by the kinetic model as FBA does not capture the feedback inhibition of acetyl-CoA on phosphoglucomutase (PGM) activity (Sanwal et al., 1972; Duckworth et al., 1973) that limits flux towards the flavanone pathway.

### Overproduction of succinate under aerobic condition

**Figure 3:**
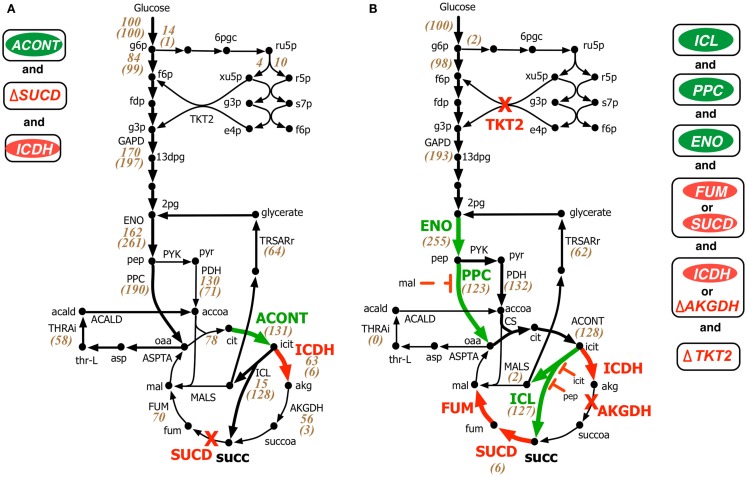
**Comparison of intervention strategies predicted by (A) regular OptForce and (B) k-OptForce for overproduction of succinate under aerobic condition in *E. coli***. The values within parentheses indicate the metabolic flux in mmol gDW^−1^ h^−1^ per 100 mmol gDW^−1^ h^−1^ glucose uptake. The values without parentheses in **(A)** show steady-state flux distribution of the reference (wild-type) strain used for kinetic model parameterization (Ishii et al., 2007).

Both OptForce and k-OptForce adopt similar strategies for re-directing flux towards succinate under aerobic condition by routing more flux through isocitrate lyase (ICL), increasing flux through phosphoenolpyruvate carboxylase (PPC), and converting the intermediate glyoxylate back to glycerate 2-phosphate (2pg) using glycerate metabolism (see Figure 3). However, the number of required interventions varies. While OptForce suggests that only three interventions are required to achieve a succinate yield of 90% of its theoretical maximum, k-OptForce suggests that additional direct up-regulations in the glycolysis and TCA cycle are necessary. For example, k-OptForce suggests at least ninefold up-regulation of ICL enzyme activity to pull TCA cycle flux from icit towards succinate. Likewise, up-regulation of enolase (ENO) enzyme by twofold of its reference activity is required to push more glycolytic flux towards succinate precursors oxaloacetate (oaa) and acetyl-CoA. Regular OptForce suggests that up-regulation of aconitase (ACONT) and down-regulation of isocitrate dehydrogenase (ICDH) are sufficient to indirectly increase flux through PPC and ICL. In contrast, k-OptForce suggests that PPC and ICL must be directly up-regulated to improve succinate yield. In addition, up-regulation of ENO pulls glyoxylate flux towards 2pg through the glycerate pathway to compensate for the pep depletion. OptForce does not require any enzymatic intervention to route metabolic flux towards acetyl-CoA sending a significant portion (58 mmol gDW^−1^h^−1^) from oaa towards acetyl-CoA using the threonine pathway. k-OptForce reveals that such a high flux cannot be routed through the threonine pathway. Even with maximum (i.e., 10-fold) up-regulation of the aspartate transaminase (ASPTA) only 20 mmol gDW^−1^h^−1^ can be diverted towards threonine. In addition, k-OptForce suggests up-regulation of PPC enzyme activity (by 50% of its reference activity) to ensure availability of equal amounts of acetyl-CoA and oaa for the production of citrate thus preventing the accumulation of intermediates.

The abovementioned interventions suggested by k-OptForce are geared towards circumventing upper bounds on max enzyme activities (i.e., no more than 10-fold). However, limits on metabolite concentrations also play a significant role in restricting flux towards succinate. The maximum yield of succinate suggested by k-OptForce (1.2 mol/mol glucose, 80% of theoretical maximum) is less than the one suggested by OptForce (1.3 mol/mol glucose, 90% of theoretical maximum). This is because as ICL is up-regulated, the concentration of intermediates pep and icit increase reaching twice their reference values. As these metabolites are competitive inhibitors of ICL, the maximum flux through the pathway towards succinate is restricted. In addition, to alleviate the regulatory effect of malate (mal) on the activity of PPC, k-OptForce also proposed a 10-fold down-regulation of the enzymes that catalyze mal production, fumarase (FUM), or succinate dehydrogenase (SUCD). Likewise, k-OptForce suggests removal of transketolase (TKT2) to alleviate the inhibition of 6-phospho-D-gluconate (6pgc) on glucose-6-phosphate isomerase (PGI) to improve the glycolytic flux towards succinate, which also reduces the production of biomass precursors.

Most of the k-OptForce interventions were consistent with engineering efforts aimed at improving succinate production under aerobic condition. For example, up-regulation of ICL and removal of SUCD and ICDH activities improved succinate yield in *E. coli* to 0.5 mol/mol glucose (Lin et al., 2005b). Further improvements in succinate production (up to 0.7 mol/mol glucose) have been achieved by up-regulation of PPC (Lin et al., 2005a). Notably, the same interventions improved aerobic succinate production in *C. glutamicum* to 0.5 mol/mol glucose (Litsanov et al., 2012). Similar to proportional up-regulation of ENO and PPC that fixes the branching ratio of the metabolic flux at pep, regulation of pep to pyruvate in the phosphotransferase system (PTS) reaction for glucose uptake was suggested to reduce the accumulation of intermediates (pyruvate and acetate) and improve succinate yield (Lin et al., 2005a). k-OptForce, however, fails to capture the accumulation of acetate upon up-regulation of PPC and glyoxylate shunt (Lin et al., 2005a; Zhu et al., 2013). This may be due to the fact that no fluxomic data for mutant strains with anaplerotic/glyoxylate shunt up-regulations was included during kinetic model parameterization. As a result, the kinetic model is unaware of the up-regulation that leads towards increased acetate production. Interestingly, k-OptForce routes glyoxylate (formed by the ICL reaction) back to 2pg using the glycerate pathway instead of the malate synthase (MALS) reaction. This pathway improves the yield of succinate since it reduces the overall loss of carbon flux to carbon dioxide. This pathway was engineered by *E. coli* (Hubbard et al., 1998; Osterhout et al., 2011) for the production of ethylene glycol and glucarate consumption, respectively, but remains to be explored for succinate overproduction.

### Overproduction of succinate under anaerobic condition

**Figure 4:**
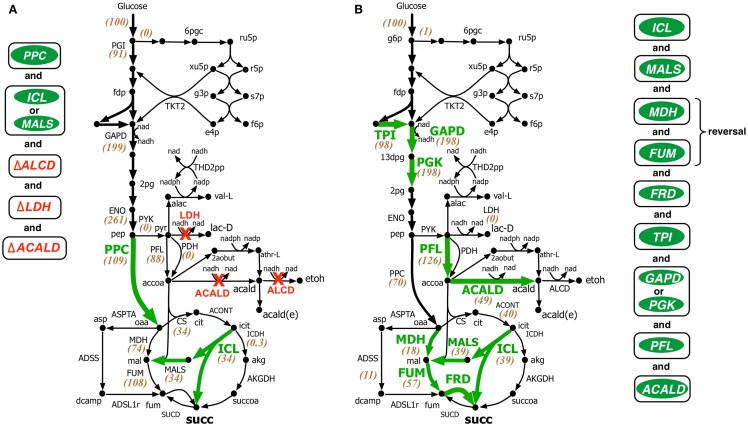
**Comparison of intervention strategies predicted by (A) regular OptForce and (B) k-OptForce for over production of succinate under anaerobic condition in *E. coli***. The values within parentheses indicate the metabolic flux in mmol gDW^−1^ h^−1^ per 100 mmol gDW^−1^ h^−1^ glucose uptake.

Under fermentative condition the electron transport chain is not active, thus preventing the oxidation of cofactor NADH generated primarily in glyceraldehyde 3-phosphate dehydrogenase (GAPD) reaction in glycolysis back to NAD. Without an adequate NADH sink, significant amount of metabolic flux is routed towards fermentative products such as ethanol, acetate, lactate, formate, etc. to restore redox balance and cellular growth. Therefore, the general strategy for succinate overproduction is to eliminate all competitive fermentative pathways while pushing more flux towards succinate through the glyoxylate shunt and reversing the reductive branch of TCA cycle (see Figure 4). This flux re-direction also regenerates NAD, thus simultaneously coupling succinate production with biomass generation.

In contrast to the aerobic case, k-OptForce suggestions for the anaerobic overproduction of succinate are less accurate compared to OptForce predictions. OptForce requires only five interventions to achieve a succinate yield of 1.42 mol/mol glucose. However, k-OptForce suggests a maximum yield of only 1.08 mol/mol glucose even after nine interventions. While k-OptForce recapitulates some of the interventions identified by OptForce (e.g., threefold up-regulation of the glyoxylate pathway enzymes ICL and MALS), the remaining suggestions deviate from OptForce and proven engineering strategies. The sources of these discrepancies can be traced back to incompatible parameterization of the kinetic model for the anaerobic case. First, due to absence of sufficient flux data in the parameterization procedure, the kinetic model was not tuned to capture reversal of the reductive branch of the TCA cycle necessary for succinate overproduction. k-OptForce suggests up-regulation of all three enzymes of the reductive branch [i.e., malate dehydrogenase (MDH), FUM, and fumarate reductase (FRD)]. However, even after a 6.5-fold up-regulation in MDH activity and 10-fold up-regulation in FUM only 80% of the anaplerotic flux (57 mmol gDW^−1 ^h^−1^) goes towards succinate, while the remaining amount (11 mmol gDW^−1 ^h^−1^) uses the aspartate metabolism to bypasses MDH and FUM (see Figure 4B).

The kinetic model also fails to capture the metabolic transition of *E. coli* central metabolism from aerobic to anaerobic condition due to lack of regulatory information (Salmon et al., 2003, 2005). Under anaerobic condition, PP pathway, PPC, and TCA cycle are repressed, while glycolysis and, in particular, fermentative pathways are up-regulated (Perrenoud and Sauer, 2005; Cho et al., 2006). In addition, pyruvate dehydrogenase (PDH) is deactivated while PFL carries most of the flux from pyruvate to acetyl-CoA (Partridge et al., 2006). Even though the kinetic model captures down-regulation of TCA cycle upon removal of oxygen it cannot capture the remaining changes. Unable to capture the repression of PPC [anaerobic PPC flux is one-tenth of aerobic flux (Choudhary et al., 2011)], k-OptForce does not suggest any up-regulation in its activity to push more flux from pep towards oaa, contrary to OptForce suggestion of a minimum 15-fold up-regulation in PPC flux (8.4–133.3 mmol gDW^−1 ^h^−1^). In contrast, failing to recognize the regulatory activation of PFL under anaerobic condition, k-OptForce suggests a minimum eightfold up-regulation in its activity, while OptForce requires no such intervention. Unable to recognize the up-regulation of the enzyme activities in the fermentative pathways in the reference (non-engineered) strain, k-OptForce does not suggest any down-regulations since the parameterization of the enzymes does not allow a significant amount of flux towards ethanol, acetate, and lactate. In contrast, OptForce requires the removal of lactate dehydrogenase (LDH), alcohol dehydrogenase (ALCD), and acetaldehyde dehydrogenase (ACALD) to prevent diverting pyruvate flux away from succinate. Surprisingly, k-OptForce suggests a fivefold up-regulation in ACALD activity to maintain NAD/NADH redox balance. A large fraction of the produced acetaldehyde is reduced to ethanol (46 mmol gDW^−1 ^h^−1^), while the rest is exported out of the cell (3 mmol gDW^−1 ^h^−1^). However, we note that as no information capturing the effect of acetaldehyde on cell fitness was included in the kinetic model, it is unable to capture the chemical’s toxicity. k-OptForce also suggests a minimum 1.5-fold up-regulation in triose phosphate isomerase (TPI) activity and a twofold up-regulation in GAPD or phosphoglycerate kinase (PGK) activity to route additional PP pathway flux through glycolysis, even though the PP pathway is negligibly active in anaerobic condition (Choudhary et al., 2011). It is to be noted here that down-regulation of TKT2 for aerobic overproduction of succinate and up-regulation of GAPD for anaerobic case are not equivalent interventions even though both strategies do increase glycolytic flux. This is because, the flux distribution in the pay-off phase of glycolysis, which is different in both cases, affects the metabolite concentrations of the preparatory phase of glycolysis. Up-regulation of ENO in aerobic overproduction study pulls additional metabolic flux down from upper glycolysis in addition to TKT2 removal. In absence of ENO up-regulation, removal of TKT2 cannot reroute the entire amount of PP flux towards glycolysis. As a result, up-regulation of both GAPD and PGK (and TPI) is necessary. It is also to be noted that the inactivation of PDH (and the subsequent activation of PFL) in anaerobic condition affects the reactions preceding it.

Comparison with experimental studies shows that unlike in the aerobic case, most of the verified engineering strategies are consistent with OptForce suggestions. k-OptForce overlooks key interventions such as up-regulation of PPC and removal of fermentative pathways, that were identified to have the largest impact in enhancing succinate yield (Millard et al., 1996; Zhang et al., 2009). In addition, even in cases where k-OptForce correctly identifies interventions, such as of MDH, FUM, and FRD up-regulation, inaccurate parameterization result in yield predictions far below experimentally observed succinate yield [1.08 vs. 1.2–1.6 mol/mol glucose with fewer interventions (Cao et al., 2013)]. In other cases, untested interventions such as up-regulation of PFL most likely will not improve succinate yield, considering that the deletion of *pflB* was found to improve succinate yield (Sanchez et al., 2005; Wu et al., 2007).

## Discussion

In this study, we compared the performance of k-OptForce in predicting interventions for overproduction of succinate in *E. coli* under both aerobic and anaerobic conditions. k-OptForce predictions under aerobic condition was found to be much more consistent with experimental strain-design strategies as compared with the stoichiometry-only OptForce predictions. In contrast, interventions for succinate overproduction under anaerobic condition by k-OptForce led to significantly less promising strategies largely inconsistent with experimental observations. This indicates that kinetic models have the potential to substantially over-perform FBA predictions when parameterized under the same (or similar) conditions but they may perform worse than FBA when asked to predict a significantly different metabolic phenotype. Note that the two-step strategy of the k-OptForce procedure does not affect the optimality of the results for the aerobic case as all interventions were identified from the kinetic part of the model. The flux distribution in the stoichiometric part of the model, which is determined by the worst-case inner problem, was effectively locked by the kinetic expressions. In general, however, we may miss better intervention strategies (for example in the anaerobic case study) when implementing the two-step approach as a tradeoff for improving computational performance.

The kinetic model was successful in capturing the underlying kinetic regulation when the flux re-distribution was consistent with the mutant flux information used for parameterizing the kinetic model. For example, the effect of enzymatic interventions around glycolysis and TCA cycle were identified with reasonable accuracy in both anaerobic and aerobic cases. Under aerobic condition, the kinetic model successfully captures the need for equimolar amounts of acetyl-CoA and oaa to supply the TCA cycle while preventing accumulation of intermediates (Lin et al., 2005a). Even when the kinetic model failed to correctly quantify fluxes, it provided a qualitative basis for making the right interventions. For example, k-OptForce correctly identifies that up-regulation of MDH, FUM, and FRD improves succinate production under anaerobic condition, even though it over-estimates the kinetic bottleneck towards such a flux-reversal resulting in poorer yields than experimental observations. Note that the developed kinetic model cannot capture changes in glucose uptake rate for different environmental and/or genetic backgrounds as all mutant fluxes used to train the model were scaled with the corresponding glucose uptake. Shortcomings in the model could be rectified by re-parameterizing the model using additional fluxomic information of mutant strains that allow for pathway reversal [e.g., using metabolic flux analysis information of a ΔSUCD strain (Li et al., 2006)]. In general, the re-parameterization is a compromise between model scope and accuracy. The observations showed that parameterizing the kinetic model by making use of mutant data located in the proximity of a target product provides a more accurate flux distribution predictions by the model and consequently results to the identification of more targeted interventions using the k-OptForce procedure. In contrast, integration of a wide-range of conditions with limited experimental data for model training may provide a better global qualitative agreement. While one could use separate kinetic models for aerobic and anaerobic conditions, ideally we would like a single model parameterization that could reproduce both aerobic and anaerobic responses. By creating two separate aerobic and anaerobic models it becomes unclear what model to use under micro/partial aerobic condition (Partridge et al., 2007).

This study shows that the model does not retain fidelity of predictions when growth is switched from aerobic to anaerobic condition. Aerobic to anaerobic metabolic transition is mainly controlled at the transcriptional level (Kochanowski et al., 2013) by the activities of global regulatory proteins FNR and ArcA (see Table 2). In absence of such regulatory interactions, the kinetic model could not capture the activation of PFL and fermentative pathways, and the deactivation of PPC and (to a small extent) PP Pathway. As a result, k-OptForce failed to identify key down-regulations (e.g., LDH, ALCD) in the former case, while suggested unnecessary up-regulations for the latter. These shortcomings are harder to address and require the incorporation of adequate regulatory information into the model (see Table 2 for details) to capture the aerobic to anaerobic transition.

**Table 2:** **Regulatory systems under anaerobic condition in *E. coli* (Partridge et al., 2006)**.

Regulator	Type	Target gene	Target reaction
ArcA	Repression	*sucABCD*	SUCOAS
		*sdhABCD*	SUCD
		*fumA*	FUM
		*mdh*	MDH
		*aceEF*	PDH
		*acnAB*	ACONT
		*gltA*	CS
		*icdA*	ICDH
	Activation	*pfl*	PFL
FNR	Repression	*acnA*	ACONT
		*icdA*	ICDH
		*sdhABCD*	SUCD
		*fumAC*	FUM
		*ndh*	NDH

*SUCOAS, succinyl-CoA synthetase; SUCD, succinate dehydrogenase; FUM, fumarase; MDH, malate dehydrogenase; PDH, pyruvate dehydrogenase; ACONT, aconitase; CS, citrate synthase; ICDH, isocitrate dehydrogenase; PFL, pyruvate formate lyase; NDH, nadh dehydrogenase*.

In general, this study revealed some of the strengths and limitations of kinetic model-driven strain design. It demonstrated the need to carry out model parameterization for a diverse range of genetic/environmental perturbations (Khodayari et al., 2014) and the tight integration of transcriptional level along with substrate-level regulatory interactions. At a fundamental level, kinetic models must be *a priori* provided with the quantitative description of as many as possible regulatory switches that become active in response to genetic or environmental perturbations. This richness in mechanistic information enables a detailed description of metabolism that captures dynamics, enzyme activities, and metabolite concentrations but can lead to erroneous predictions due to missing and/or incorrect modeling assumptions. Nevertheless, by studying failure modes of kinetic models, valuable information can be uncovered for restoring prediction consistency for new phenotypes.

## Author Contributions

Conceived and designed experiments: Costas D. Maranas, Anupam Chowdhury, Ali Khodayari. Performed the experiments: Anupam Chowdhury and Ali Khodayari. Analyzed the data: Anupam Chowdhury, Ali Khodayari, Costas D. Maranas. Contributed reagents/materials/analysis tools: Anupam Chowdhury, Ali Khodayari, Costas D. Maranas. Wrote paper: Ali Khodayari, Anupam Chowdhury, Costas D. Maranas.

## Conflict of Interest Statement

The authors declare that the research was conducted in the absence of any commercial or financial relationships that could be construed as a potential conflict of interest.
